# Mechanistic Analysis of Reflective Cracking Potential in Electrified Pavement with Inductive Charging System

**DOI:** 10.3390/ma17174282

**Published:** 2024-08-29

**Authors:** Pengyu Xie, Hao Wang

**Affiliations:** Department of Civil and Environmental Engineering, School of Engineering, Rutgers, The State University of New Jersey, Piscataway, NJ 08854, USA

**Keywords:** electrified pavement, finite element modeling, pavement design, reflective cracking, inductive charging

## Abstract

Electrified pavements with inductive charging systems provide an innovative way of providing continuous wireless power transfer to electric vehicles (EVs). Electrified pavements have unique construction methods, resulting in different mechanical and thermodynamic characteristics from traditional pavements. This study aimed to investigate the mechanistic design of electrified pavements to mitigate thermal-induced reflective cracking due to the inclusion of concrete slabs with inductive charging units (CUs) under an asphalt surface layer. Finite element (FE) models were developed to analyze the temperature profiles, pavement responses, and crack potential in electrified pavements. The fatigue model and Paris’ law were utilized to evaluate crack initiation and propagation for different pavement designs. Within the allowable range for sufficient wireless charging efficiency, increasing the surface layer thickness had a noticeable benefit on mitigating crack initiation and propagation. The results indicate that increasing the asphalt surface layer thickness by 20 mm can delay crack initiation and propagation, resulting in a two to threefold increase in the number of cycles needed to reach the same crack length. Reflective cracking can also be retarded by the optimized design of the charging unit. Increasing the concrete slab thickness from 100 mm to 180 mm resulted in an approximately 20% increase in the number of cycles to reach the same crack length. Reducing the slab width and length (shortening joint spacing) could also effectively reduce the reflective cracking potential, with the slab length having a more significant influence. These findings highlight the importance of balancing charging efficiency and structural durability in the design of electrified pavements.

## 1. Introduction

Electric vehicles (EVs) are an efficient solution for reducing greenhouse gases (GHGs) and enhancing the sustainability of the transportation system [1]. EVs show better energy efficiency than traditional vehicles with internal combustion engines due to the usage of more efficient power trains and electric motors [2]. Instead of using fossil fuels like gasoline or diesel, lithium-ion batteries are widely employed in EVs due to the high energy density, high efficiency, long lifespan, and high potential of improvement [3]. However, the charging speed and battery capacity limit the travel distance of EVs, especially for heavy trucks. Therefore, new techniques need to be developed to charge EVs to expand the driving range.

Dynamic charging can supply power to the EV while it is in motion. Dynamic charging functions either by conductive or inductive (wireless) charging, depending on whether the vehicles are physically connected to the charging unit [4]. When compared to conductive charging, inductive charging offers several advantages in terms of simplicity, reliability, and user-friendliness [5]. Generally, the energy is transferred from the primary coil, which is embedded in the pavement structure, to the secondary coil, which is mounted underneath the vehicle [6]. However, the larger distance between the primary coil in the pavement and the secondary coil in the vehicle leads to increased leakage inductance and magnetizing current, consequently diminishing the energy transfer efficiency. This restriction limits the embedded depth of the charging unit that may affect the integrity of the pavement structure and result in premature distresses.

A few pilot projects have been implemented to construct an electrified pavement with the ability of inductive charging [7,8,9]. These projects have primarily explored the efficiency of energy transfer between the charging units embedded in the pavements and vehicles. This has resulted in a lack of a comprehensive understanding of how the inductive charging system affects the pavement structural integrity and field performance. It is expected that pavements with an embedded inductive charging system will perform differently from traditional pavements. These differences are primarily influenced by changes in the pavement structure, the increased temperature resulting from the charging operation, and the truck loading on the charging lane. These thermal and mechanical effects may impact the stress distribution in the electrified pavement, and accordingly, its performance [10].

The construction of electrified pavements can be conducted on both flexible and rigid pavements. The charging lane can be reconstructed with rigid pavements with CUs inside concrete slabs. This option has the advantage of utilizing common concrete slabs through in situ construction or prefabrication techniques [11,12], but it requires full lane reconstruction at high costs. On the other hand, flexible pavements are preferred due to the ease of construction as rehabilitation requires relatively low costs. For example, trenches can be excavated in the existing asphalt pavement to install small-sized concrete slabs incorporating CUs, and then the asphalt surface layer is placed. Nevertheless, pavement distress can arise due to the introduction of bi-material interfaces and localized mechanical discontinuities or poor compaction due to construction difficulty.

The pavement distresses caused by the inclusion of CUs is the primary concern for the design of electrified pavements from the aspect of pavement integrity [13]. A few studies have been conducted to evaluate the impact of charging units on pavement rutting and fatigue cracking. Chabot and Deep indicated that the smaller modulus ratio between the prefabricated concrete slab incorporating CUs and the top layers could mitigate pavement failure [14]. Chen et al. found that the potential of surface-related damage or interface deboning was accelerated for flexible pavements by considering that the presence of a charging lane would cause more braking and accelerating behavior and the lane changing of electric trucks [15]. It was found that the surface layer above the inductive charging unit was the weakest location in the pavement structure [9,11]. Proper modification of the pavement surface will help to expand the lifespan of electrified pavements such as adding additive polymer materials [16,17,18].

To improve the charging efficiency, concrete slabs with inductive CUs inside can be placed close to each other with joints, similar to concrete slabs in composite pavements. Traffic-induced reflective cracking may not be critical since the charging unit does not need to cover the whole lane and can only be placed in the middle of the lane where wheel loading is at its minimum. Instead, thermal-induced reflective cracking becomes the major concern due to the horizontal movement and curling of concrete slabs under changing temperatures. There have been no studies on how the design and installation of inductive CUs may affect the reflective cracking of electrified pavements. Therefore, further research is needed to evaluate the pavement responses and damage mechanism of electrified pavements with inductive CUs.

## 2. Objective and Scope

This study aimed to investigate the mechanistic design of electrified pavements with an inductive charging system. The specific construction method for electrified pavements focused on embedding concrete slabs with charging units in the asphalt pavement. Finite element models were developed to predict the temperature distributions and calculate the stress and fracture mechanic parameters for analyzing the initiation and propagation of thermal-induced reflective cracking in the studied electrified pavement system. The design parameters including the embedment depth and dimension of charging units were analyzed to identify the design factors in mitigating the development of reflective cracking.

## 3. Development of Finite Element Model

### 3.1. Structure of Electrified Pavement

**Figure 1:**
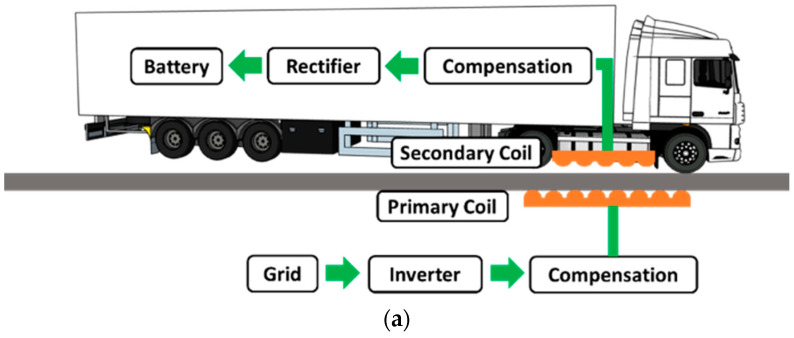

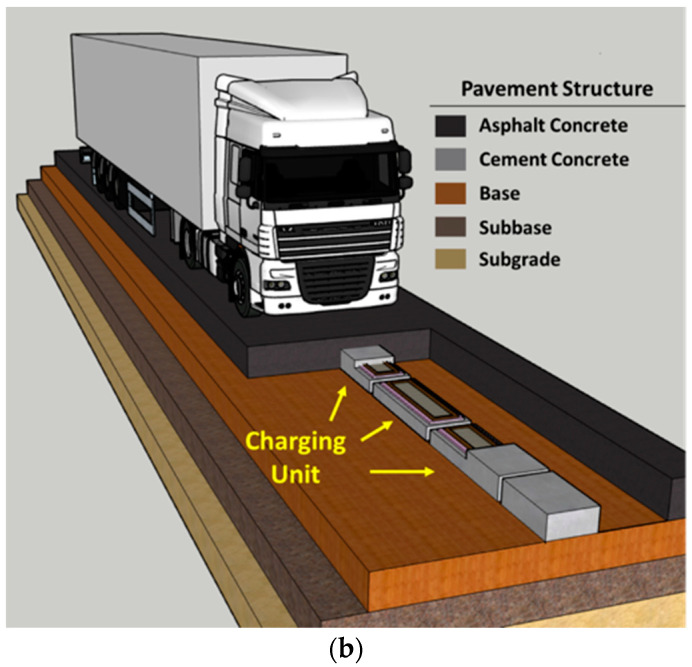
Illustration of (**a**) an electrified roadway with an inductive charging system and (**b**) pavement design with embedded charging units.

Figure 1a depicts the design concept of an electrified roadway incorporating an inductive charging system for EVs. A charging unit (CU) was embedded in the pavement with the assembly of a primary coil and compensation circuit. The prevalent technology for wireless power transfer is inductive coupled power transfer (ICPT), which transfers power via alternating magnetic fields between primary and secondary (receiver) coils connected with capacitors [19]. The proposed electrified pavement structure embedded charging units (CUs) in concrete slabs that were located in the middle of the lane and covered with an asphalt surface, as depicted in Figure 1b. Electrified pavements can be constructed by milling the existing surface, then excavating a trench for the installation of concrete slabs with charging units, and finally applying a thin asphalt surface layer on the top of concrete slabs. For new pavements, the CUs can be placed without trenching, followed by the construction of the surrounding asphalt layer and surface layer. The embedment of concrete slabs with CUs alters the pavement integrity, and it is important to maintain good bonding between the concrete slabs and surrounding asphalt mix using tack coats. If the CUs are placed close to each other to maximize the charging power, the concrete slab joints can result in reflective cracking in the asphalt surface layer. Since the inductive charging unit is placed in the middle of the lane, traffic loading will not be directly applied on the transverse joints of concrete slabs due to the small width of the charging unit. Therefore, thermal-induced cracking is the major cause of pavement failure.

### 3.2. Development of Finite Element Models

Finite element (FE) models of the electrified pavement were developed to evaluate thermal-induce reflective cracking. The models can be categorized into two parts: the first model employs field climate data to calculate temperature profiles in the electrified pavement. Next, the temperature variation is incorporated into the mechanical model as thermal loading to determine the critical pavement responses for analyzing the initiation and propagation of reflective cracking. Specifically, the initial condition of the mechanical model is set to the high temperature during the temperature variation period. The analysis process corresponds to the cooling phase, which is the most critical condition for thermal-induced reflective cracking.

**Figure 2:**
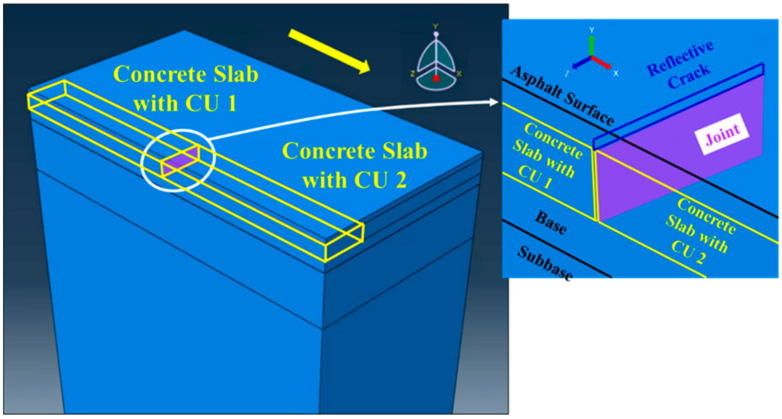
Schematic diagram of the 3D FE electrified pavement model.

The developed pavement model contains six components including the asphalt surface layer on top of the charging unit, concrete slab with charging unit, existing asphalt layer, granular base, granular subbase, and subgrade, as shown in Figure 2. To save on computational time, only a half lane was modeled with the symmetrical plane along the longitudinal direction of the driving direction. The baseline of the computational domain was 1850 × 3500 × 3790 mm in width, length (traffic direction), and thickness. Alternative pavement structures were analyzed to find the optimized electrified pavement design by changing the layer thicknesses and CU dimensions. In the thermal model used to calculate the pavement temperature, all side and bottom faces were thermally insulated, allowing for energy exchange between the pavement structure and the environment solely through the pavement surface. For the mechanical model, the bottom of the subgrade was fixed in all directions, and lateral constraints were applied to the sides of the model. These boundary conditions have commonly been used in previous studies [20].

Two charging units formed a 6.35-mm wide joint underneath the asphalt surface layer. The initial crack was considered as a “through-width” crack across the charging unit and the crack propagation was assumed to only be upward. The cracks were simulated using the extended finite element method (XFEM), which employs enrichment functions to capture the singularity at the crack tip and the displacement discontinuity across the crack surface. As a result, the model simplifies the meshing process and allows the crack to propagate arbitrarily rather than along a predefined path [21]. Equation (1) shows the approximation for the displacement vector function with the partition of unity enrichment in XFEM [21]. On the right side of the equation, the first term, second term, and third term were valid for all nodes, the nodes whose shape function support was cut by the crack interior, and nodes whose shape function support was cut by the crack tip, respectively [21].

(1) $$u=\sum_{I=1}^{N}N_{I}\left(x\right)\left[u_{i}+H\left(x\right)a_{i}+\sum_{a=1}^{4}F_{\alpha }\left(x\right)b_{I}^{\alpha }\right]$$

where $N_{I}\left(x\right)$ are the usual nodal shape functions; $u_{i}$ is the usual nodal displacement vector associated with the continuous part of the FE solution; $a_{i}$ and $b_{I}^{\alpha }$ are the nodal enriched degree of freedom vector; $H\left(x\right)$ is the discontinuous jump function across the crack surface; $F_{\alpha }\left(x\right)$ represents the elastic asymptotic crack-tip functions.

Based on the preliminary models, the mesh size in an asphalt overlay impacts the convergence of the pavement responses, especially the fracture potential indicators like stress intensity factors and J-integral. Therefore, refined meshes were utilized in regions close to the crack, while the coarse meshes were employed in areas farther away. Refined mesh and coarse mesh were seamlessly connected using a “tie connection” in the simulation, which is commonly used to tie two separate parts together to avoid relative motion between them. A mesh sensitivity analysis was conducted for refined mesh. The results showed that using 2–4 mm elements in the longitudinal/vertical directions and 40 mm elements in the transverse will balance both the model accuracy and computation time. The coarse mesh area used gradually increasing element sizes in both the transverse and longitudinal directions based on their distance from the joint. Element sizes ranged from 40 mm to 80 mm in the transverse direction and from 10 mm to 150 mm in the longitudinal direction. More detailed model development has been illustrated in previous studies and can be referenced as needed [20,22].

### 3.3. Climate Inputs and Temperature Prediction

The temperature profiles in electrified pavements were predicted by the FE thermal models. The energy interaction between the pavement structure and ambient environment was conducted through three mechanisms, which were short-wave radiation (solar irradiance), long-wave radiation (pavement emission), and convection. The governing equations for these mechanisms are written in Equations (2)–(5).

(2) $$q_{abs}=\left(1-\alpha \right)\times q_{solar}$$

(3) $$q_{conv}=h\times (T_{s}-T_{a})$$

(4) $$q_{irr}=\varepsilon \times \sigma \times (T_{s}^{4}-T_{sky}^{4})$$

(5) $$h=5.678\left(1.3+1.135U^{0.75}\right)$$

where $q_{abs}$, $q_{conv}$, $q_{irr}$ are the heat flex through short-ware radiation, convection, and long wave radiation, respectively; α is the albedo of asphalt surface; $q_{solar}$ indicates the solar radiation that reaches the pavement surface; h is the coefficient of convection of the AC surface, which is highly influenced by wind speed U; ε is the emissivity of the asphalt surface; *σ* is the Stefan–Boltzmann constant, which equals 5.67 × $10^{8}$ ($Wm^{-2}K^{-4}$); $T_{a}$, $T_{s}$ and $T_{sky}$ are the air temperature, surface temperature, and sky temperature.

The short-wave radiation was modeled by the “surface heat flux” command in ABAQUS, which is considered as a type of external thermal loading applied to the pavement surface. This model requires two key factors: solar irradiance and albedo. The former can be obtained from the LTPP database, which indicates the solar energy reaching the pavement surface. The latter, set as 0.15, determines the fraction of solar energy the pavement system absorbs. By incorporating these two parameters, the developed FE model can accurately determine the short-wave radiation from the solar energy.

The heat convection was modeled in ABAQUS with the command “surface film condition”. Three key factors influenced this heat transfer mode including the surface temperature ($T_{s}$), air temperature ($T_{a}$), and coefficient of convection (h). During the heat transfer analysis, $T_{s}$ can be determined from the pavement temperatures results of the previous time step, $T_{a}$ is the climate input, h is also calculated from the climate input wind speed (U), which is then imported into the model. With all of this information, the heat convection procedure can be calculated by the proposed model.

The long-wave radiation was modeled using the “surface radiation” command, which is influenced by four parameters: surface temperature ($T_{s}$), sky temperature ($T_{sky}$), emissivity (ε), and the Stefan–Boltzmann constant (σ). The sky temperature ($T_{sky}$) represents the temperature of the blackbody radiation that has the same flux of the downward atmospheric radiation and can be determined by using the air temperature and dew point temperature. The climate input, humidity, is used to calculate the dew point temperature. The emissivity (ε) was set to 0.9, a typical value for an asphalt surface. The Stefan–Boltzmann constant equaled 5.67 × $10^{8}$ ($Wm^{-2}K^{-4}$). $T_{s}$ can also be derived from the pavement temperature results of the previous time step. Thus, all of these parameters were then input into the thermal FE model to calculate the long-wave radiation.

Model validation was conducted in earlier work to verify the accuracy of the developed thermal model. Site-specific climate data and pavement temperature data were extracted from the Long-Term Pavement Performance (LTPP) database. Two pavement sections under summer and winter climate conditions were selected to compare the measured and calculated pavement temperatures. The summer data came from section 7023 in Maine, with pavement layers of 155 mm AC overlay, 196 mm PCC slabs, and a 394 mm base layer. The winter data came from section 3013 in Ohio, with layers of 89 mm AC overlay, 208 mm PCC slabs, and a 102 mm base layer. The results demonstrate that the developed thermal simulation model consistently produced temperature profiles that matched the measurements. Further details on pavement temperature modeling can be referred to elsewhere [22].

**Figure 3:**
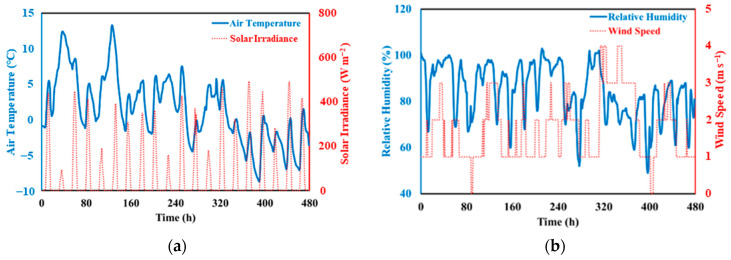
Climate inputs of the thermal FE model. (**a**) Air temperature and solar irradiance and (**b**) relative humidity and wind speed.

The climate inputs were the hourly-based winter climate data between 27 November and 16 December 2018 in New York City, NY. The initial temperature of the pavement structure was expected to significantly influence subsequent pavement temperatures. Therefore, a simulation period of 20 days prior to the study period was conducted to eliminate the impact of the initial temperature. Solar irradiance, air temperature, wind speed, and relative humidity were extracted from the Long-Term Pavement Performance (LTPP) database and employed in the developed FE model. Figure 3a,b illustrates the typical climate inputs utilized in this study. To predict the initiation and growth of reflective cracking under thermal loading cycles, one typical cooling process was extracted from the study period, in which the air temperature decreased from 0.1 °C to −5.8 °C over a period of 19 h. In this case, the purpose of analysis was not to accurately predict the pavement life, but to analyze the influences of the design parameters for the reflective cracking potential.

### 3.4. Pavement Material Properties

**Figure 4:**
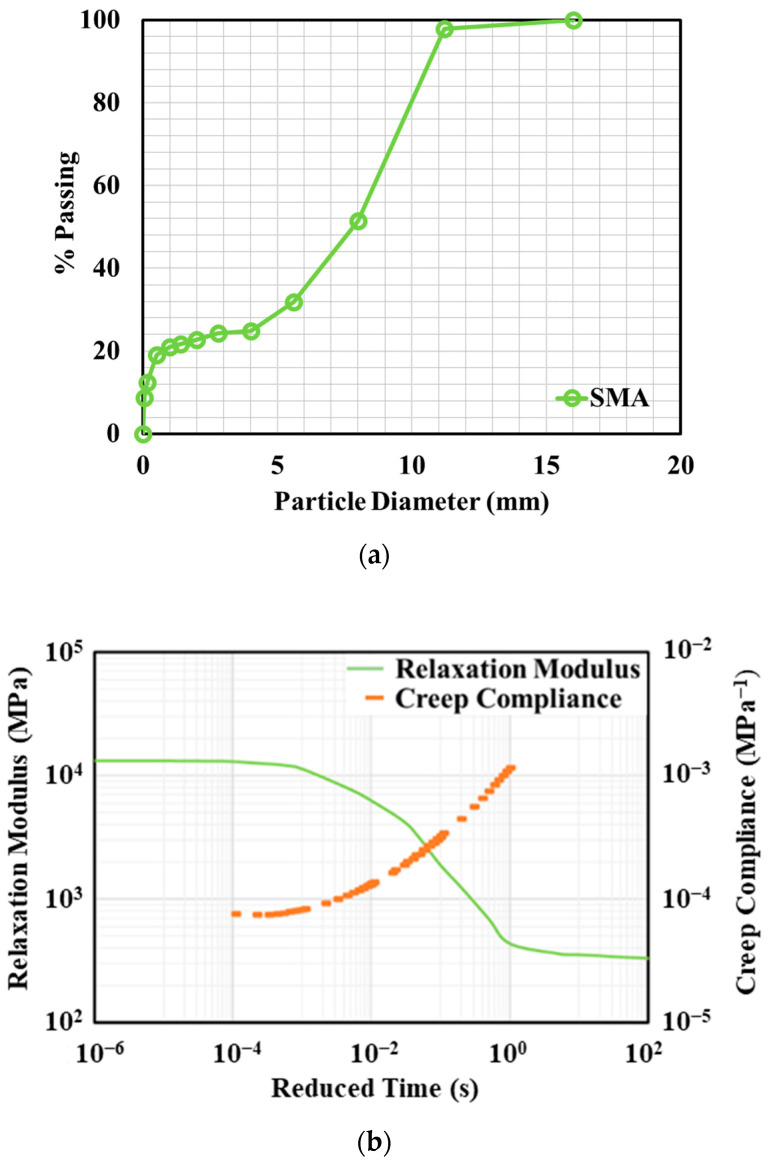
Material properties of asphalt concrete (SMA). (**a**) Gradation, (**b**) creep compliance, and relaxation modulus [25].

The asphalt surface and existing asphalt layer surrounding the concrete slabs were treated as a viscoelastic material, while the concrete slab, base, subbase, and subgrade were considered as elastic materials. Considering the thin-surface layer of the electrified pavement, a special type of asphalt concrete, stone mastic asphalt (SMA), was used for the asphalt surface and existing asphalt layer. SMA features a stone-on-stone skeleton to enhance the mixture’s strength, and its abundant mortar binder contributes to its durability. In comparison to traditional dense-graded asphalt mixtures, SMA exhibits a superior resistance to rutting and cracking [23,24]. The material properties of SMA were from laboratory experiments by Jacobs [25]. The binder type was bitumen 80/100 and the gradation is shown in Figure 4a. The creep compliance and relaxation modulus are presented at the reference temperature of 15 °C, as shown in Figure 4b.

For the viscoelastic properties of the asphalt surface layer, the bulk (K) and shear (G) moduli were derived from the relaxation modulus (E) and fitted into a generalized Maxwell solid model, as described in Equations (6)–(8) [26]. Equation (9) represents the Williams–Landell–Ferry (WLF) function, which quantifies the horizontal shift at varying temperatures through shift factors [21]. The other mechanical and thermal material properties were obtained from the literature for common pavement materials, as shown in Table 1 [27].

(6) $$E\left(t\right)=\frac{sin\left(n\pi \right)}{n\pi D\left(t\right)}$$

(7) $$G\left(t\right)=G_{0}\left[1-\sum_{i-1}^{N}G_{i}\left(1-e^{-t/\tau_{i}}\right)\right]$$

(8) $$K\left(t\right)=K_{0}\left[1-\sum_{i-1}^{N}K_{i}\left(1-e^{-t/\tau_{i}}\right)\right]$$

(9) $$log\left(a_{T}\right)=-\frac{C_{1}\left(T-T_{0}\right)}{C_{2}+\left(T-T_{0}\right)}$$

where *E* indicates the relaxation modulus; *D* represents the creep compliance; *n* is the power value of the local pure power law expression; *t* represents the relaxation time; *G*_0_, *K*_0_ are the instantaneous shear and volumetric elastic modulus, respectively; *G_i_*, *K_i_*, *τ_i_* are the Prony series parameters; N indicates the number of terms in the equation; e is the base of the natural logarithm; $a_{T}$ is the shift factor; *T*_0_ represents the reference temperature; *T* is the temperature; *C*_1_ and *C*_2_ are the regression coefficients in the WLF function.

### 3.5. Crack Initiation and Propagation

Fatigue cracks emerge as the pre-existing imperfections evolve and coalesce under cyclic loading. The crack initiation phase is defined as the very beginning of crack development and its fracture mechanism is different from the macro-propagation of cracks. Cracks in the asphalt mixture have been evaluated in well-designed fatigue tests and the transformation threshold from micro imperfections to macro cracks was characterized as 7.5 mm [28]. The total fatigue cycle is correlated to the material responses (stress and strain) in the form of the power law. To capture the trend close to the real thermal loading situation, the stress-control four-point bending fatigue tests at low temperature and low loading frequency were utilized to derive the fatigue model, as shown in Equation (10) [29].

(10) $$N_{i}={\left(\frac{S_{v}}{\sigma }\right)}^{n^{\prime }}$$

where *N_i_* represents the fatigue life; σ represents the stress at fatigue test; *n*′ is the constant that represents the material property and varies at different temperatures (2.596 at 0 °C, 3.114 at −10 °C, and 3.241 at −20 °C used here as an example). $S_{v}$ represents the strength of the asphalt concrete, which was selected as 3 MPa for low frequency loading due to thermal stress.

The Paris–Erdogan law is the most widely used mechanical model to estimate micro crack development under cyclic loading. Instead of using the stress intensity factor to indicate the fracture status at the crack tip, the J-integral is employed considering the viscoelasticity of the asphalt material [30], as shown in Equations (11) and (12). The fracture parameters (A and n) were determined from the derived Gaussian process regression model [20]. Instead of conducting time-consuming fatigue tests, this model linked the common material characteristics (such as mixture modulus, binder content, and temperature) of asphalt concrete with fracture parameters.

(11) $$J=\int \Gamma \left(wdy-T_{i}\frac{\partial u_{i}}{\partial x}ds\right)$$

(12) $$\frac{da}{dN}=A\cdot {\left(∆J\right)}^{n}$$

where Γ is an arbitrary counterclockwise path around the crack tip; w is the strain energy density; ∂x and dy are length increments along the *x* and *y* direction, respectively; $T_{i}$ represents components of the traction vector; $u_{i}$ indicates the displacement vector components; ds is the length increment along the contour Γ; da/dN indicates the crack increment after unit cycle; ΔJ is the variation of the J-integral; A and n indicate the fracture properties of asphalt concrete.

The proposed crack growth models were validated by comparing the full-scale reflective cracking test conducted at the National Airport Pavement Test Facility (NAPTF). A two-strip overlay structure with different thicknesses (76 mm and 152 mm) was constructed to investigate the growth of reflective cracking under the temperature-effect simulation system (TESS). Horizontal displacements were applied to the PCC slabs to simulate thermal expansion and contraction of the rigid layer. The calculated crack initiation and propagation cycles were consistent with the test results, validating the feasibility of using the proposed model to predict the growth of reflective cracking. More details about the model validation can be referred to in previous work [20]. 

### 3.6. Modeling of Layer Interfaces

The interface between the asphalt surface layer and concrete slab was characterized by the cohesive interface model with the traction–separation law, which enabled us to simulate how the interface bonding materials (e.g., tack coat) resisted separation and ultimately failed. This model works well in modeling bi-material interfaces such as composite pavements [31]. The interface stiffness primarily influences the relative displacement of the asphalt surface and concrete slabs under thermal loading. Previous research has calibrated the shear stiffness of 1.4 MPa/mm in the cohesive model using full-scale tests of composite pavements [20]. On the other hand, the Coulomb friction model can be utilized for the interfaces between concrete slabs, granular base/subbase, and subgrade. This friction coefficient has minimal effect on reflective cracking.

## 4. Temperature Profiles in Electrified Pavement

**Figure 5:**
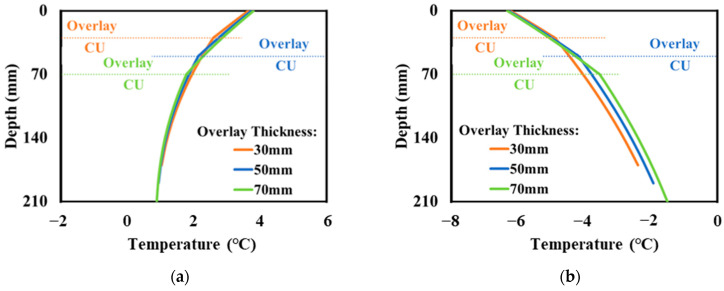
Temperature profiles of the electrified pavements at (**a**) the beginning and (**b**) end of the temperature drop period.

Due to the inclusion of the charging unit, the temperatures profiles of electrified pavements were different from those in traditional flexible pavements. This study comprehensively evaluated the pavement temperature profiles with the consideration of different design parameters for electrified pavements including the overlay thickness and all CU dimensions. It was found that the effect of charging unit dimensions on temperature was not significant compared to the effect of the asphalt surface layer thickness. Figure 5a,b shows the temperature distribution for electrified pavements with different overlay thicknesses, respectively, at the beginning and the end of the temperature drop period. The “CU” indicates concrete slabs with charging components in the electrified pavement. “Overlay” represents the new asphalt overlay after installing the charging units.

Since concrete has a higher thermal conductivity, the embedment of CUs results in higher temperature gradients in electrified pavements, suggesting a more rapid temperature decrease in the CUs of the concrete slab compared to the asphalt overlay. Figure 5a shows that the temperature profile remained relatively unchanged with the variation in overlay thickness at the beginning of the temperature drop period. Figure 5b illustrates that varying the overlay thickness had a limited impact on the temperature within the overlay material, whereas the temperature in the CU underwent noticeable changes after experiencing the temperature drop. The increase in the overlay thickness from 30 mm to 70 mm postponed the rapid temperature decreasing trend in the CU, leading to a higher slab temperature. Therefore, a smaller joint opening can be expected for a thicker asphalt overlay, which may reduce the reflective cracking potential.

It is noted that the charging operation may increase pavement temperatures due to power loss in the form of heat [32]. Reflective cracking tends to occur at low temperatures or after experiencing significant cooling processes. Higher temperatures soften the asphalt mixture and reduce the contraction of the concrete slab, possibly causing it to expand slightly. This would increase the fracture resistance of the asphalt mixture and reduce the joint opening of the concrete slab. Thus, the heat generated from the CU was neglected here to consider the highest potential of reflective cracking.

## 5. Parametric Study of Electrified Pavement Design

Basically, the electrified pavement design intends to balance the charging efficiency and pavement deterioration, depending on the embedment depth and dimension of the charging units. The thinner asphalt surface is beneficial for the charging efficiency due to the smaller distance between two inductive coils, while the pavement is prone to reflective cracking. Thus, several important design parameters of electrified pavement were analyzed in this study to quantify their influences on thermal-induced reflective cracking including the embedment depth, thickness, and width of concrete slabs with CUs. The recommendation of jointed plain concrete pavements from the American Concrete Pavement Association was used to determine the length (join spacing) of the concrete slabs based on its thickness [33]. Table 2 summarizes the structure parameters of the electrified pavement analyzed in this study. Since electrified pavements are still in the stage of a pilot project, parametric analysis is helpful for understanding the impact of an inductive charging system on pavement response and damage evolution.

### 5.1. Effect of Embedment Depth of Charging Unit

It is desirable to embed CUs close to the pavement surface to minimize the energy loss. Therefore, compared with traditional flexible pavements, the asphalt surface layer of electrified pavements is thin. To ensure the high efficiency of wireless power transfer (WPT), the distance between primary and receiver coils is recommended to be smaller than 400 mm [34]. Considering the typical clearance under EVs is 200 to 300 mm from the ground, three embedment depths of charging units were considered in the analysis including 30 mm, 50 mm, and 70 mm.

**Figure 6:**
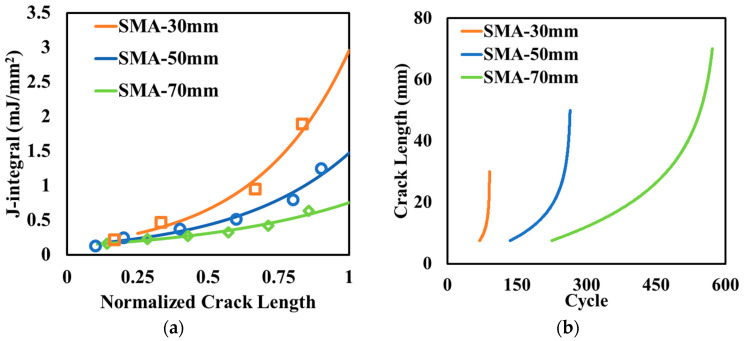
Effect of thickness of the asphalt surface layer on (**a**) the variation in the J-integral and (**b**) crack length.

Figure 6a shows how the J-integral changed at various crack lengths. Since the thicknesses of the asphalt surface layer were different, the crack length was divided by the asphalt thickness to obtain the normalized crack length and presented as the x-axis in the figure. The J-integral was first calculated from the numerical simulation model, and then the regression models were developed to capture the J-integral, avoiding time-consuming calculation. As the crack propagated upward, the J-integral increased exponentially, which means that cracks grow more. This trend aligns with observations from the field experiments, where reflective cracking exhibited slow initial growth, gradually accelerating as it approached the pavement surface [35].

Meanwhile, with the increase in the surface thickness, the crack potential decreased significantly, even though the increase interval as relatively small (20 mm). On the one hand, a thicker surface works as “thermal isolation” to reduce the temperature drop in concrete slabs, which limits its contraction in developing thermal-induced reflective cracking. On the other hand, the thicker surface increases the confining pressure at the crack tip, helping restrict crack growth. Figure 6b compares the calculated crack length versus loading cycles for different surface thicknesses. It indicates that the thicker surface layer in electrified pavements could potentially extend the pavement life. For example, a 20-mm thickness increase in the asphalt surface layer led to a two to threefold increase in the number of cycles to reach the same crack length.

It should be noted that the thicker surface layer increase in the distance gap between the primary and secondary coils in the wireless charging system led to a reduction in the charging efficiency nonlinearly [19,36]. Therefore, the optimized design of the asphalt surface thickness balancing the pavement life and charging efficiency needs be considered in future study.

### 5.2. Effect of Charging Unit Width

**Figure 7:**
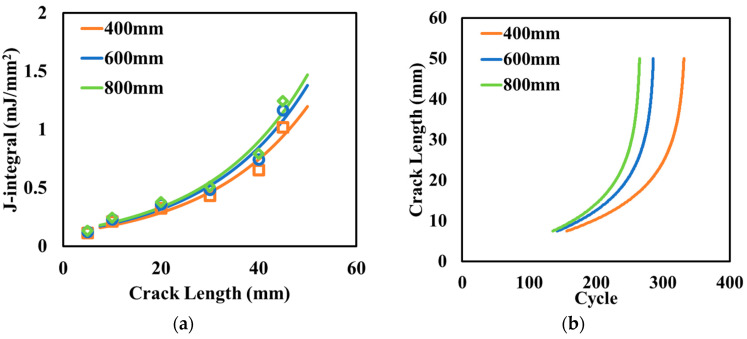
Effect of the width of the concrete slab with CUs on (**a**) the variation in the J-integral and (**b**) crack length.

The dimensions of the concrete slabs with CUs may vary with the design of the primary coil and compensation circuit. Three widths were considered in this section, which were 400 mm, 600 mm, and 800 mm. Figure 7a shows the J-integral at different crack lengths. The reduction in the slab width mitigated the crack potential in the asphalt surface layer. Because the contraction of the concrete slab was the primary reason for the thermal-induced reflective cracking, the narrower slab reduced the total tension applied at the bottom of the asphalt surface layer, weakening the joint opening effect induced by the cooling process. Figure 7b shows the crack length with the thermal cooling cycles for crack initiation and propagation. The reduction in the slab width showed larger cycle numbers for both the crack initiation and propagation phase, with the number of loading cycles to reach the same crack length increasing to 1.25 times the original one when the charging unit width decreased from 800 mm to 400 mm.

In the proposed design of electrified pavements, concrete slabs are embedded in the middle of the lane to transfer energy to EVs. Under normal driving conditions, concrete slabs with smaller widths increase the distance away from tire loading, which further helps reduce the load-induced stress on the concrete slab with CUs. This indicates that reducing the width of the charging unit can extend the pavement service life while protecting the electric components.

### 5.3. Effect of Charging Unit Thickness

**Figure 8:**
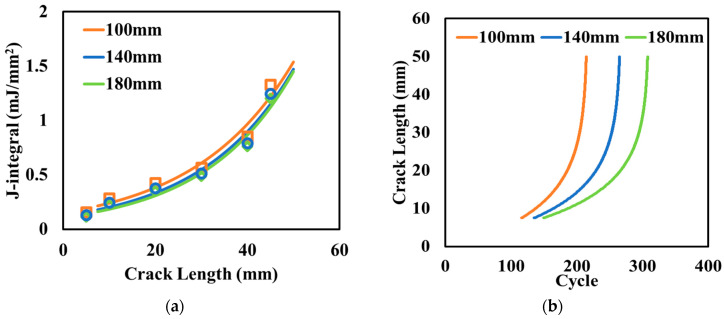
Effect of the thickness of the concrete slab with CUs on (**a**) the J-integral and (**b**) crack length.

Three different thicknesses of concrete slabs with CUs were considered in this section: 100 mm, 140 mm, and 180 mm. Figure 8a illustrates that the J-integral decreased slightly as the thickness of concrete slab increased, which indicates that the thicker slab had a smaller reflective crack potential in asphalt surface layer. At the same depth in the electrified pavement structures with difference slab thicknesses, the temperatures in the surface layer and concrete slab with CUs were almost the same. If the thicker slab was considered as a combination of the upper part (same thickness of thin slab) and lower part (extra part of thicker slab), the upper part experienced the same temperature drop as the thinner slab and would generate the same contraction. However, during the cooling process, the pavement temperature trend was to increase as the depth went down. Thus, the lower part has a higher temperature and less contraction than the upper part. The continuity of concrete slabs makes the lower part a constraint for the upper part and results in smaller overall contraction. Therefore, the thicker concrete slab with CUs would result in smaller joint openings than that of thinner slabs, and thus has less reflective cracking potential in the asphalt surface layer. During the cooling process, the maximum joint openings before crack initiation were 0.130 mm, 0.135 mm, 0.142 mm, if the thicknesses of the concrete slabs were 180 mm, 140 mm, and 100 mm, respectively. The trend of joint opening varying with slab thickness was consistent with the analytical results on the temperature of the concrete slab.

The calculated crack length with loading cycles for different slab thicknesses are plotted in Figure 8b. Increasing the 40-mm slab thickness resulted in a slight increase in both the crack initiation and propagation cycles, and the total number of cycles to reach the same crack length increased by around 20%. Therefore, thicker concrete slabs with CUs are better at mitigating thermal-induced reflective cracking in electrified pavements.

### 5.4. Effect of Charging Unit Length

**Figure 9:**
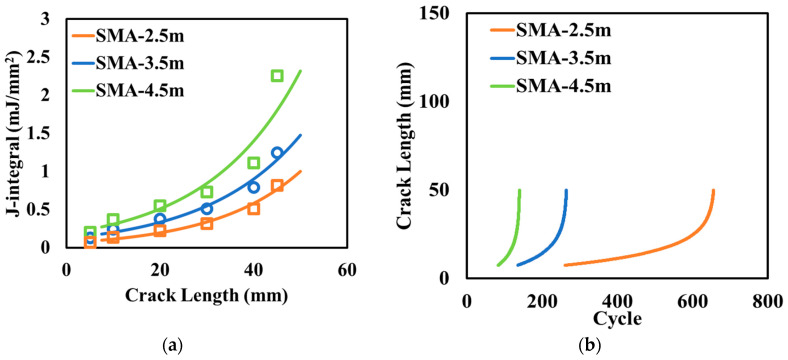
Effect of joint spacing on (**a**) the variation of the J-integral and (**b**) crack length.

The transverse joint is a key feature to prevent the thermal cracking generated by the contraction of concrete. The concrete slabs with CUs should also have joints to relieve the stress induced by thermal contraction. Three different lengths of concrete slabs (joint spacing) were evaluated in the analysis: 2.5 m, 3.5 m, and 4.5 m. Figure 9a plots the variation of the J-integral with crack length and Figure 9b illustrates the growth of the crack length under cyclic loading. The smaller joint spacing results in less reflective cracking potential. When the joint spacing decreases from 4.5 m to 2.5 m, the number of loading cycles to reach the same crack length increases by 3.5 times.

During a cooling process in winter seasons, long joint spacing (slab length) tends to have a wider joint opening, which aggravates the development of reflective cracking. However, smaller joint spacing may increase the difficulty of construction and generate more reflective cracking from the whole pavement length point of view due to the increased number of joints.

## 6. Conclusions and Recommendations

Electrified pavements can be constructed by embedding concrete slabs with inductive charging units under an asphalt surface layer. The study analyzed thermal-induced reflective cracking in an electrified pavement using finite element models with fracture mechanics. The effects of various design parameters on the performance and durability of electrified pavements were evaluated.

The asphalt surface layer is relatively thin in an electrified pavement because high wireless charging efficiency requires a primary coil and secondary coil within a short distance. The asphalt surface layer has noticeable effects on the temperature condition responsible for thermal-induced reflective cracking in the electrified pavement. Increasing the asphalt layer thickness by 20 mm can delay crack initiation and propagation, resulting in a two to threefold increase in the cycles needed to reach the same crack length. On the other hand, the reflective cracking of an electrified pavement can be mitigated by the optimized design of concrete slabs with charging units. Thicker concrete slabs with charging units show less potential for reflective cracking. Specifically, a 40-mm increase in slab thickness enhanced the crack initiation and propagation cycles by approximately 20%. This was attributed to smaller joint openings during temperature cooling, which mitigates the stress concentration at the joints. Narrower and shorter concrete slabs also reduce the tension at the bottom of the asphalt layer and thus retard the development of reflective cracking. The number of loading cycles to achieve the same crack length increased by a factor of 3.5 when the joint spacing was decreased from 4.5 m to 2.5 m, highlighting the importance of optimized joint design in crack mitigation. These findings provide scientifically significant insights into the design of electrified pavements. Quantifying the impact of design parameters on cracking behavior enables more informed decision making for future pavement designs.

Future work is recommended to focus on balancing the charging efficiency and structural durability for electrified pavements. The optimized design of electrified pavements will be conducted regarding two aspects. For an inductive charging system, coil configurations and ferrite cores can be designed to improve the power transfer efficiency. Meanwhile, the mitigation measures of cracking in asphalt surface layer can be explored such as a stress-absorbing interlayer or surface mix with high fracture resistance.

## Figures and Tables

**Table 1:** Pavement material properties.

		Existing Asphalt/Surface Layer	Concrete with CU	Granular Base	Granular Subbase	Subgrade
	
Density (kg·m^−3^)		2300	2401	2200	2100	1800
Modulus (MPa)		Viscoelastic	27500	350	200	69
Poisson’s Ratio		0.35	0.15	0.35	0.35	0.4
Conductivity (W m^−1^K^−1^)		1.3	2.5	1.56	1.43	1.56
Specific heat (kJ kg^−1^K^−1^)		0.92	0.88	0.91	0.94	1.04
Expansion coefficient (°C^−1^)		2 × 10^−5^	0.98 × 10^−5^	n/a	n/a	n/a
Fracture parameters—A		1.91 × 10^−6^	n/a	n/a	n/a	n/a
Fracture parameters—n		2.035	n/a	n/a	n/a	n/a

**Table 2:** Structure parameters of electrified pavement.

Charging Lane Width (mm)		3700
Asphalt surface over concrete slabs	Thickness (mm)	30, 50, 70
Concrete slabs with charging units	Thickness (mm)	100, 140, 180
	Width (mm)	400, 600, 800
	Joint spacing (mm)	2500, 3500, 4500
Layers below concrete slabs	Base layer thickness (mm)	100
	Subbase layer thickness (mm)	500
	Subgrade layer thickness (mm)	3000

## Data Availability

Data will be made available on request.
